# Some Remarks on Mr. Smith's Statistical Inquiry Respecting the Frequency of Stone in the Bladder in Great Britain and Ireland

**Published:** 1821-04

**Authors:** Cornelius Tripe

**Affiliations:** Member of the Royal College of Surgeons in London.


*Some Remarks on Mr. Smith's Statistical Inquiry respecting the frequency of Stone in the Bladder in Great Britain and Ireland.* By Cornelius Tripe, Member of the Royal College of Surgeons in London.

IN the first part of the eleventh volume of the Medico-Chirurgical Transactions, there appears a statistical inquiry into the frequency of stone in the bladder in Great Britain and Ireland, by Richard Smith, Esq. senior surgeon of the Bristol Infirmary.

The substance of this paper, as it relates to the frequency of the disease, has been derived from the different county hospitals through the United Kingdom, in consequence of circulars, which were addressed by Mr. Smith to the surgeons of those establishments upon this subject. The information thus derived is erroneous to a very great extent.

Mr. Barnes, one of the surgeons of the Devon county hospital, in replying, for himself and his colleagues, to the inquiry which was addressed to the surgeons of that establishment, states the number of calculous patients which have been submitted to operations in that hospital, in the interval between 1806 and 1816, to be sixteen or eighteen cases; in the interval between 1811 and 1818, nine cases. Mr. Barnes also observes as follows, "This hospital may be considered as embracing *very nearly all* the stone-cases amongst the poor; for in this county, and some part of the adjoining counties of Somerset, Dorset, and particularly Cornwall, operations for the stone out of the hospital *are very rare.*" He says again, "The population of Devon, in 1811, was 383,000: one may fairly calculate the hospital to have within its circle the stone-operations, with very few exceptions, of a population of 600,000; the population of Cornwall exceeding 200,000."

Within the last ten years, I have, in my private practice, operated for the stone on thirteen subjects of various ages; one of whom was a female, aged twelve years. The greater number of these patients were incapable of rendering any pecuniary return for the operation; and all of them were cured, excepting my last case, a boy twelve years old, who died in forty-eight hours after the operation, of consecutive inflammation. I have very lately sounded two male children, and shall, according to every probability, ere long operate on them. I have also known, within the last ten years, of two male adults who have died of the disease, without any operation having been performed: the existence of the disease was proved by dissection.

Mr. Hammick, the principal surgeon of the Royal Naval Hospital, whose name must be sufficiently familiar to the profession, and from whom (I feel a pride in stating it,) I derived the earliest principles of my professional education, first drew my attention to this subject a few days since. He, having witnessed, with my other friends, all my operations, was, in consequence, struck with the inaccuracy of Mr. Barnes's report, which he had then just read in the volume which has been referred to. He has also requested me to state, that he is quite certain there has been in these towns, within the last ten years, a number of cases of stone performed on by different surgeons, which, taken together, quite equal those which I have enumerated in my practice.

Thus it will appear, that the number of stone-operations in the private practice of an individual, during the last ten years, as far as we can judge by Mr. Barnes's report, is equal to the number of cases which have been operated on at the county-hospital within the same period. Add to this the cases operated on by other surgeons in these towns, and the total will then double the number which have been operated on in that hospital.

To say the least of Mr. Barnes's report, it exhibits a deficiency of necessary caution in reporting for the whole county of Devon, as he has done, without previously instituting an inquiry on the subject, of some one or other of those professional gentlemen who are practising in so important a part of the county as this is. The towns of Plymouth Dock, and Stonehouse, being very near each other, contain together about 70,000 inhabitants; and they are surrounded by an extensive country, spreading into Cornwall, from whence diseases which are of an extraordinary character are constantly arriving, to obtain professional assistance. Had he condescended to make this inquiry, he would never have assigned, as he has done, to the institution of which he is a surgeon, the almost exclusive merit of treating this disease; stating that the Devon hospital embraced nearly all the stone-cases for the counties of Devon, Cornwall, &c. and that such operations any where, except in that hospital, were extremely rare in those counties.

May not some of the reports which have been furnished from other places be marked with similar want of caution? I would therefore submit, with deference, the impossibility, under the view of the error which has been taken, and the suspicion which such a circumstance is capable of casting over the reports in general, of any just opinions or inferences being drawn from the inquiry, as it at present stands.

I beg I may not be misunderstood as insinuating a suspicion that any individual who has contributed to that inquiry could be guilty of intentional omission. But it appears to me, that the surgeons of county-hospitals may not be aware to what extent the improved science of surgery has become diffused, in the present day, over the face of the country; and therefore I would suggest that hereafter, if information be required by any member of the profession, who may be actuated with such laudable motives as Mr. Smith has evinced, in addition to the mode of inquiry which he has adopted at the different county-hospitals, an invitation should be inserted, in the periodical Journals, to the profession in general, to contribute the results of their experience on the subject required.

I will only add, that, instead of the opinion prevailing here that the disease has become more rare, the question is frequently asked, "How is it that the stone in the bladder has become so frequent an occurrence, compared to former times?" We answer, that, to the best of our belief, the disease is not more frequent now than it has hitherto been, but that such cases were formerly not noticed, or were sent to the county-hospitals for cure; and hence, also, probably arises the difference in the comparative number of cases which we have seen published from that institution.

*Plymouth Dock; February 14th, 1821.*

